# Glycolysis-related radiosensitivity signature for predicting radiotherapy response in breast cancer

**DOI:** 10.3389/fimmu.2025.1638897

**Published:** 2025-10-02

**Authors:** Xuan Lin, Shiyin Hu, Linyan Huang, Tianwen Xu, Jinzhi Lai

**Affiliations:** ^1^ The Second Affiliated Hospital of Fujian Medical University, Quanzhou, Fujian, China; ^2^ Department of Ultrasound, First Affiliated Hospital of Xiamen University, School of Medicine, Xiamen University, Xiamen, Fujian, China; ^3^ Department of Oncology, The Second Affiliated Hospital of Fujian Medical University, Quanzhou, Fujian, China

**Keywords:** breast cancer, glycolysis, radiosensitivity, PD-L1, tumor immune microenvironment

## Abstract

**Background:**

This study investigates the influence of glycolytic activity on the efficacy of radiotherapy in breast cancer (BRCA) patients, aiming to develop a glycolysis-related radiosensitivity signature to predict radiotherapy efficacy.

**Methods:**

We categorized BRCA patients into low and high glycolysis groups using ssGSEA analysis based on 200 glycolysis-related genes. Univariate and multivariate Cox regression analyses, were used to construct a radiosensitivity signature. Immune cell infiltration and pathway enrichment analyses were conducted using ESTIMATE and CIBERSORT methods. The TIDE algorithm and pRRophetic algorithm were employed to predict responses to radiotherapy. Radioresistant BRCA cells were examined using CCK-8 assay. Key genes identified in the radiosensitivity signature were validated *in vitro* by qRT-PCR. Seahorse assay was used to evaluate cellular glycolytic capacity.

**Results:**

Our analyses revealed that patients in the low-glycolysis group exhibited enhanced sensitivity to radiotherapy, suggesting that glycolytic activity is a critical determinant of radiotherapy. Subsequently, we developed a four-gene radiosensitivity signature that effectively stratified patients into radiosensitive (RS) and radioresistant (RR) groups. Survival analysis revealed that radiotherapy significantly improves outcomes in the RS group but not in the RR group. Immune infiltration analysis indicated that the RS group correlates with an active immune landscape, as evidenced by lower TIDE scores and higher responsiveness to immune checkpoint inhibitors. Notably, patients in the RS group with high PD-L1 expression showed significantly better outcomes, associated with increased immune cell infiltration. *In vitro* validation using MCF-7 and radioresistant MCF-7/IR cell lines confirmed that radioresistant MCF-7/IR cells exhibit increased glycolytic activity.

**Conclusion:**

Our study establishes glycolytic activity as a promising predictor of radiotherapy efficacy in BRCA patients and develops a novel radiosensitivity signature with potential clinical utility in guiding personalized treatment strategies.

## Introduction

Breast cancer (BRCA) is one of the most prevalent malignancies affecting women worldwide and remains a leading cause of cancer-related morbidity and mortality (1). Despite advances in therapeutic strategies such as surgery, chemotherapy, and targeted therapies, the optimal management of breast cancer remains challenging, particularly in patients with advanced stages of the disease (2, 3). Radiotherapy (RT) is a cornerstone of breast cancer treatment, utilized not only as an adjuvant therapy following surgery but also as a primary treatment in inoperable cases (4). However, a significant clinical challenge persists: the variability in patient responses to radiotherapy, with some patients experiencing substantial benefits while others demonstrate resistance (5). This underscores an urgent need for predictive models that can accurately stratify patients based on their likelihood of responding to radiotherapy, thereby optimizing individualized therapeutic approaches.

Glycolysis, the metabolic pathway responsible for converting glucose into pyruvate, is often upregulated in tumors, a phenomenon commonly referred to as the Warburg effect (6). This preferential use of anaerobic glycolysis for energy production is associated with enhanced tumor growth, proliferation, and, noteworthy in our context, radiosensitivity (7). Growing evidence suggests that glycolytic activity can modulate tumor responses to radiotherapy by influencing DNA repair mechanisms and oxidative stress levels, particularly under hypoxic conditions prevalent in tumor microenvironments (8). Research has suggested that high glycolytic activity may alter tumor behavior, either promoting or inhibiting responses to therapies depending on the tumor microenvironment and metabolic state (9). Given this crucial link, glycolysis-related molecular features hold promise as predictive biomarkers for radiotherapy efficacy (10). Leveraging glycolytic pathway activity to stratify breast cancer patients may therefore improve identification of radiosensitive and radioresistant phenotypes, guiding clinical decision-making.

Recent advances in high-throughput sequencing technologies have revolutionized our understanding of tumor biology and the development of predictive models (11, 12). These techniques permit comprehensive profiling of genomic and transcriptomic landscapes, enabling the identification of biomarkers associated with treatment sensitivity and resistance (13). A variety of research studies have utilized both cellular and animal models to investigate alterations in gene and protein activity after exposure to radiation, with the goal of uncovering a reliable molecular profile that can forecast how sensitive cancer cells are to radiotherapy (14–16). As an example, Torres-Roca and colleagues created a 10-gene-based radiosensitivity index (RSI), which demonstrated strong predictive power for the radiation response across 48 different cancer cell lines (17). Kim et al. identified a 31-gene signature from NCI-60 microarray data that serves as a significant prognostic tool for BRCA patients receiving radiotherapy (18). Building on prior research, our study aims to establish a robust prognostic model integrating glycolysis-related gene expression to enhance the prediction of radiotherapy sensitivity in breast cancer patients.

In this study, we analyzed transcriptomic datasets to characterize the relationship between glycolytic activity and radiotherapy response in breast cancer. We constructed and validated a radiosensitivity signature based on glycolysis-related genes, which effectively stratifies BRCA patients into radiosensitive (RS) and radioresistant (RR) groups according to their radiosensitivity index, thereby identifying those who derive the greatest survival benefit from radiotherapy. Functional pathway elucidation, genomic landscape characterization, and immune profiling further revealed mechanistic insights into radiosensitivity phenotypes. Importantly, this signature demonstrated potential clinical utility by predicting outcomes of radiotherapy and other anti-tumor therapies, offering a valuable tool for precision oncology in breast cancer management. By identifying patients likely to benefit from radiotherapy, we may improve clinical management of breast cancer, paving the way for enhanced therapeutic precision in this prevalent disease.

## Materials and methods

### Data collection and processing

Gene expression profiles along with corresponding clinical data for BRCA patients were obtained from two publicly accessible repositories: The UCSC Xena platform (https://xena.ucsc.edu/) and The Molecular Taxonomy of Breast Cancer International Consortium (METABRIC) database (http://www.cbioportal.org/) (19, 20). For the TCGA-BRCA dataset, data were downloaded in Fragments Per Kilobase Million (FPKM) format and converted to TPM for downstream analysis. The METABRIC dataset underwent preprocessing through robust multi-array average (RMA) and quantile normalization techniques to maintain data uniformity. To ensure integrity and comparability, inclusion criteria were applied as follows: (1) only primary breast cancer samples were considered; (2) patients required complete clinical follow-up data with a minimum duration exceeding 30 days; (3) detailed radiotherapy treatment information had to be available. Following these filters, 928 cases from TCGA-BRCA and 1980 cases from the METABRIC cohort meeting the criteria were selected for subsequent analyses, each with matching RNA-sequencing data and clinical details.

To assess glycolytic activity, we utilized the HALLMARK_GLYCOLYSIS gene set (200 genes) from the Molecular Signatures Database (MSigDB), a curated and widely validated hallmark pathway for glycolytic metabolism (21). This gene set was selected due to its comprehensive coverage of core glycolytic enzymes and regulatory networks. The ssGSEA algorithm, which quantifies pathway enrichment at the single-sample level, was applied to calculate the absolute glycolysis activity score for each patient. Patients were stratified into high and low glycolysis subgroups based on the median ssGSEA score within each cohort for downstream analyses.

### Radiosensitivity signature development using glycolysis-related genes

For the purpose of developing a radiosensitivity signature, we included patients from the TCGA-BRCA cohort. To identify glycolysis-related genes that significantly correlate with overall survival (OS) in patients receiving radiotherapy while showing no association in non-radiotherapy patients, we conducted univariate Cox regression analysis. Following this, a multivariate Cox regression analysis was applied to refine the selection of prognostic GRGs and construct a radiosensitivity signature (22). The resulting signature is derived using the following formula, which incorporates the expression levels of selected genes along with their respective coefficients to calculate a radiosensitivity index (RSI) for each patient:


$$\begin{array}{l} Radiosensitivity\;index=(Expression\;of\;Gene\;1\times Coefficient\;of\;Gene\;1)+\\ (Expression\;of\;Gene\;2\times Coefficient\;of\;Gene\;2)+\\ \cdot \cdot \cdot +(Expression\;of\;Gene\;n\times Coefficient\;of\;Gene\;n) \end{array}$$

Each patient was then categorized into either the RS or RR subgroup based on the median RSI value. The RS group comprises patients predicted to show improved OS due to radiotherapy when compared to non-radiotherapy counterparts. In contrast, no appreciable survival benefit from radiotherapy was observed among patients classified into the RR group, regardless of their treatment status.

### Functional pathway enrichment and immune cell infiltration analysis

To uncover the differences in cellular pathways between the RS and RR groups, we implemented Gene Set Variation Analysis (GSVA) for Kyoto Encyclopedia of Genes and Genomes (KEGG) pathway enrichment analysis (23). This identified the molecular pathways most significantly enriched in each group. Additionally, we utilized Gene Ontology (GO) enrichment analysis to provide a deeper insight into the biological processes (BP), molecular functions (MF), and cellular components (CC) associated with each group. These analyses provide a comprehensive view of the functional divergence underlying differential radiosensitivity in breast cancer.

To characterize the tumor immune microenvironment and assess patterns of immune cell infiltration in the RS and RR groups, we applied the ESTIMATE algorithm to infer the presence of infiltrating immune and stromal cells (24). This method generates three scores for each sample: immune score, stromal score, and overall ESTIMATE score, which reflect the relative abundance of immune and stromal components within the tumor tissue. In parallel, we used the CIBERSORT computational method to deconvolute the composition of tumor-infiltrating immune cells, estimating the proportions of 22 distinct immune cell subtypes (25). Only those samples with a CIBERSORT-derived p-value < 0.05 were included in subsequent analyses. The resulting immune cell fractions were normalized so that their sum equaled one, allowing for proportional comparisons across samples. Following this, ssGSEA was performed to evaluate the enrichment levels of 29 immune-related signatures, which reflect various immune functions and pathways (23).

### Prediction of immunotherapy, chemotherapy and targeted-therapy response

To evaluate the potential responsiveness of breast cancer patients to immunotherapy, we utilized the Tumor Immune Dysfunction and Exclusion (TIDE) scores, which were accessed from the TIDE portal (http://tide.dfci.harvard.edu/) (26). This approach integrates transcriptomic profiles to calculate TIDE scores, along with separate scores for T cell dysfunction and T cell exclusion, which collectively reflect the probability of resistance or responsiveness to immune checkpoint blockade. Furthermore, we retrieved Immunophenotype scores (IPS) relevant to CTLA-4 and PD-1 blockade from The Cancer Immunome Atlas (TCIA) database (https://tcia.at/home) (27). These scores are derived from key immune-related gene expression signatures and are scaled from 0 to 10, with higher values indicating greater potential for effective antitumor immune activation in response to immunotherapy.

For estimating responses to conventional chemotherapeutic agents and targeted therapies, we employed the pRRophetic R package (28), which predicts drug sensitivity based on gene expression profiles. This tool uses baseline sensitivity data from the Genomics of Drug Sensitivity in Cancer (GDSC) database to infer the half-maximal inhibitory concentration (IC_50_) values for a range of commonly used anticancer drugs, thereby enabling the stratification of patients according to their predicted therapeutic benefit.

### Cell culture and establishment of radioresistant BRCA cells

The human breast cancer cell line MCF-7 was obtained from the American Type Culture Collection (ATCC, Manassas, USA, Lot Number: 70019550). Both MCF-7 and its radioresistant derivative, MCF-7/IR, were maintained under identical conditions in Minimum Essential Medium (MEM), supplemented with 10% fetal bovine serum (FBS; Corning, USA) and 1% penicillin-streptomycin solution (Gibco-BRL, USA). All cell cultures were incubated at 37 °C in a humidified atmosphere containing 5% CO_2_. To generate the radioresistant cell line, MCF-7 cells were exposed to fractionated γ-irradiation. A total cumulative radiation dose of 60 Gy was delivered in 2 Gy fractions, five days per week over six weeks. Control cultures underwent identical handling procedures without radiation exposure, serving as the non-irradiated reference group. The development of radio-resistance was confirmed through cell viability assays. Briefly, both MCF-7 and MCF-7/IR cells were seeded into 96-well plates and exposed to single doses of 0, 4, or 8 Gy of γ-radiation. Following irradiation, cells were incubated with CCK-8 reagent according to the manufacturer’s instructions. Absorbance at 450 nm was measured using a microplate reader, with optical density values reflecting relative cell viability post-treatment.

### Quantitative real-time polymerase chain reaction

Total RNA was isolated from cultured cells using TRIzol^®^ reagent (Invitrogen, San Diego, CA, USA) in accordance with the manufacturer’s guidelines. RNA quantity and purity were evaluated using a NanoDrop 2000 spectrophotometer (Thermo Fisher Scientific, USA), ensuring optimal integrity for downstream applications. Gene expression levels were determined via qRT-PCR, using the SYBR^®^ PrimeScript™ RT-PCR Kit (Invitrogen, USA) as per the manufacturer’s instructions. Gene-specific primer pairs are detailed in **Supplementary Table S1**. Each reaction was run in triplicate alongside no-template controls to confirm the absence of contamination or nonspecific amplification. The threshold cycle (Ct) values were normalized to the average Ct values of reference genes, with GAPDH serving as the primary internal control. Relative mRNA expression was calculated using the 2^^−ΔΔCt^ method and expressed as fold change relative to control samples.

### Measurement of extracellular acidification rate using seahorse assay

To evaluate cellular glycolytic capacity, ECAR was measured using the XF96 Extracellular Flux Analyzer (Seahorse Bioscience). All assay media and reagents were prepared according to the manufacturer’s recommendations. MCF-7 and MCF-7/IR cells were seeded into XF96 cell culture microplates at a density of 5 × 10^4^ cells per well. Following overnight attachment, the growth medium was removed and replaced with fresh assay medium supplemented with specific metabolic modulators. The ECAR assay and OCR assay were measured using a Seahorse with Seahorse XF Glycolytic Rate Assay Kit (Seahorse Bioscience) following the manufacturer’s instructions. ECAR values were normalized to the cell number in each well to account for any potential variations in cell density.

### Statistical analysis

All statistical evaluations were carried out using R software (version 4.1.3). The selection of appropriate statistical tests was based on the type and distribution of the data. The Chi-square test was utilized for comparisons involving categorical variables or pairwise features across different groups. For assessing statistically significant differences between two groups, the Mann-Whitney U test was employed. When comparing categorical variables or pairwise features among multiple independent groups, the Kruskal-Wallis test was applied. To evaluate linear relationships between normally distributed variables, Pearson’s correlation coefficient was calculated. Spearman’s rank correlation coefficient was used to analyze non-parametric data that displayed non-normal distributions. Kaplan-Meier curves were produced to examine differences in survival outcomes between two or more groups, with the log-rank test applied to evaluate statistical significance. Unless otherwise stated, all hypothesis tests were two-sided, and a p-value < 0.05 was considered statistically significant. This conventional threshold was used consistently across all analyses to maintain uniformity and interpretability.

## Results

### Glycolytic activity predicts radiotherapy efficacy in BRCA patients

**Figure 1:**
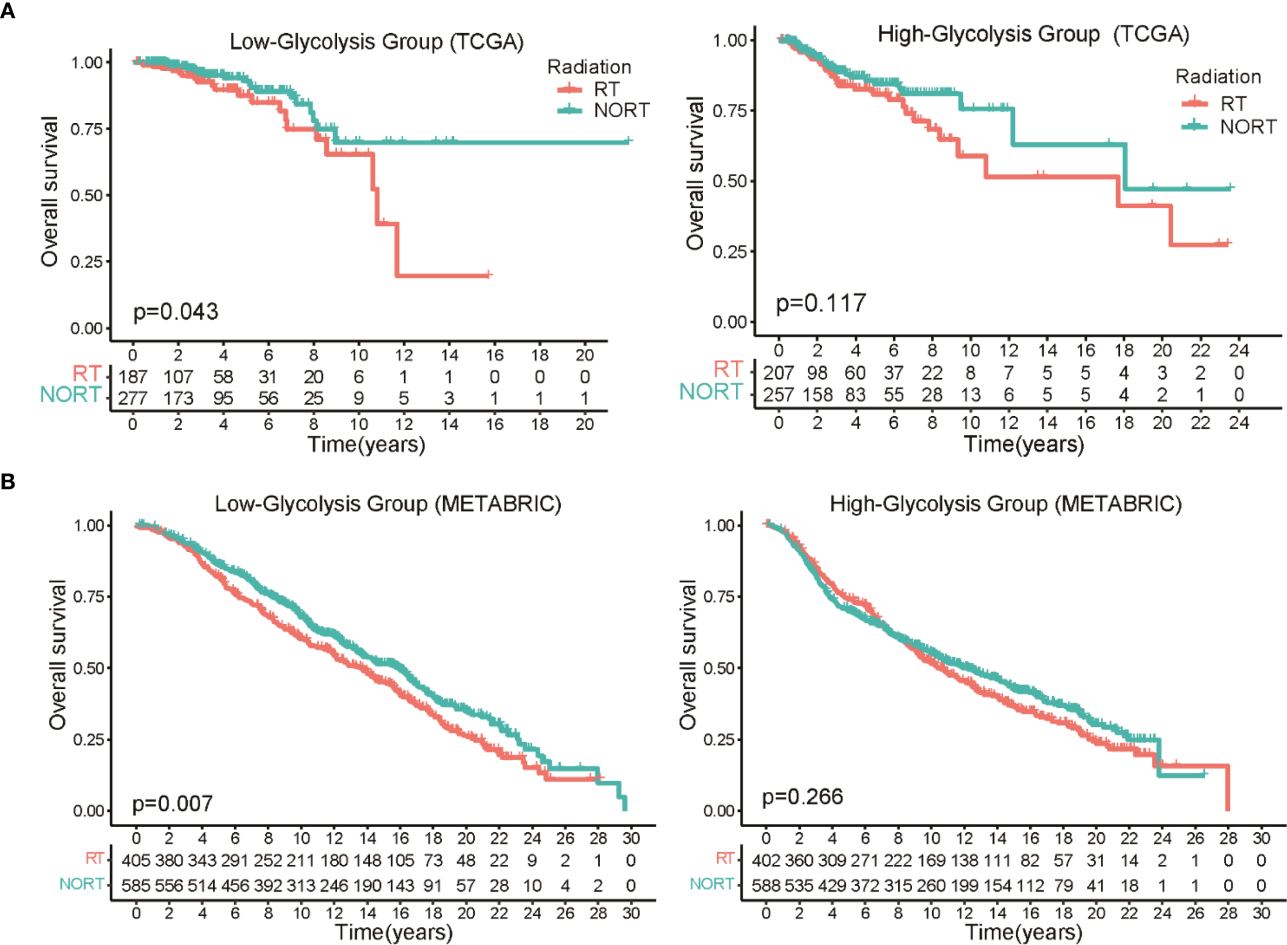
Association between glycolytic activity and radiotherapy response in BRCA patients. **(A)** The Kaplan-Meier survival curves clearly demonstrate a significant survival advantage for patients with low glycolysis levels who underwent radiotherapy, while no such benefit was observed in the high glycolysis group. **(B)** Survival analysis in the METABRIC dataset show prolonged OS in low glycolysis patients who underwent radiation therapy versus those who did not.

The methodological pipeline for this study is outlined in **Supplementary Figure S1**. Initially, we categorized TCGA-BRCA patients into low-glycolysis and high-glycolysis groups using the ssGSEA algorithm, leveraging 200 glycolysis-related genes from the MSigDB database. The survival analysis across all BRCA patients showed no significant link between OS and glycolysis scores (**Supplementary Figure S2A**). However, a distinct pattern emerged when radiotherapy outcomes were evaluated according to glycolysis levels. In the low-glycolysis group, patients undergoing radiotherapy had significantly better OS than non-radiotherapy patients. In contrast, in the high-glycolysis subgroup, no notable difference in survival was seen between treated and untreated individuals (**Figure 1A**). To validate this observation, we applied the same classification criteria to the METABRIC dataset. The survival analysis indicated that in the low-glycolysis group, RT patients had significantly better OS than non-radiotherapy patients (**Figure 1B**). These data imply that glycolytic activity may influence how BRCA tumors respond to radiotherapy. Principal component analysis (PCA) showed that the glycolysis related genes could clearly categorize BRCA patients into two groups (**Supplementary Figure S2B**). Further analysis of the relationship between glycolysis activity and clinicopathological features in TCGA-BRCA patients revealed no significant differences in clinical stage, age distribution, or menopausal status between the two groups (**Supplementary Figure S2C**). Yet, the high-glycolysis group had a significantly higher proportion of overweight individuals than the low-glycolysis group.

### Development of a radiosensitivity signature linked to glycolysis in BRCA patients

**Figure 2:**
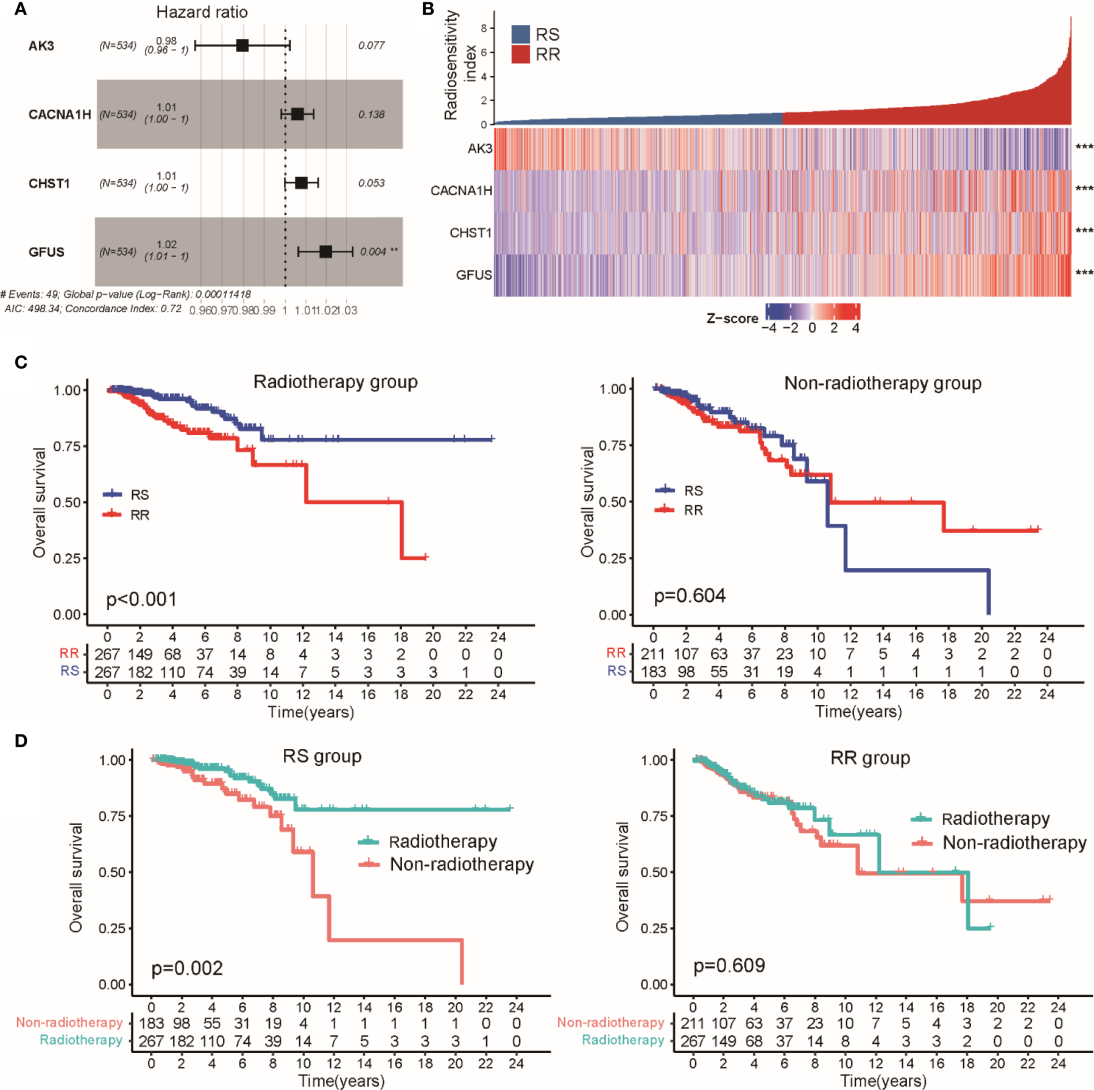
Establishment of a radiosensitivity signature linked to glycolysis in BRCA patients. **(A)** Multivariate Cox regression results are presented in a forest plot, showing the hazard ratios and 95% confidence intervals for each gene included in the radiosensitivity signature. **(B)** The heatmap and color-coded matrix illustrate the expression patterns of the four genes constituting the radiotherapy response index between RS and RR groups. **(C)** Kaplan-Meier survival curves comparing OS between radiotherapy and non-radiotherapy patients within the RS and RR groups. **(D)** Kaplan-Meier plots displaying OS differences according to radiosensitivity classification, separated by radiotherapy treatment status. ** p<0.01, *** p<0.001.


To evaluate the radiosensitivity of BRCA patients within the TCGA-BRCA cohort, we developed a signature associated with glycolysis-related gene expression. Initially, univariate Cox regression was conducted on 200 glycolysis-related genes across three groups: patients receiving radiotherapy, those not receiving it, and the entire cohort (**Supplementary Figure S3A**). This analysis identified nine glycolysis-related genes significantly associated with OS in both the radiotherapy group and the entire cohort, but not among the patients who did not undergo radiotherapy (**Supplementary Figure S3B**). Subsequently, multivariate Cox regression was used to create a radiosensitivity signature specific to radiotherapy patients, resulting in a model incorporating four genes (**Figure 2A**). The radiosensitivity index (RSI) was generated by the formula:

$$\begin{array}{l} RSI=(expression\;of\;AK3\times -0.0207)+(expression\;of\;CACNA1H\times 0.0059)+\\ (expression\;of\;CHST1\times 0.0079)+(expression\;of\;GFUS\times 0.0195). \end{array}$$

Applying the median value of this index to all TCGA-BRCA cases, patients were divided into RS and RR groups (**Figure 2B**). Survival analysis showed that within the RS group, patients treated with radiotherapy had significantly better OS than those who did not receive radiotherapy. In contrast, the RR group showed no significant difference in OS regardless of radiotherapy (**Figure 2C**). Moreover, among patients who underwent radiotherapy, the RS group had a notably better OS compared to the RR group, while no OS difference was observed between these groups in patients who did not receive radiotherapy (**Figure 2D**). Similar trends were observed when analyzing disease-specific survival (DSS) and progression-free interval (PFI), where radiotherapy was linked to improved outcomes specifically in the RS group (**Supplementary Figures S3A–B**). We extended the validation to the METABRIC-BRCA dataset by applying the same RSI formula to classify patients. Consistently, Kaplan-Meier curves demonstrated superior OS for radiotherapy-treated patients classified as RS relative to other subgroups (**Supplementary Figure S2C**). Collectively, these findings suggest that the proposed glycolysis-related radiosensitivity signature may serve as a valuable predictor of radiotherapy efficacy in breast cancer.

### Functional pathway analysis related to radiosensitivity groups

**Figure 3:**
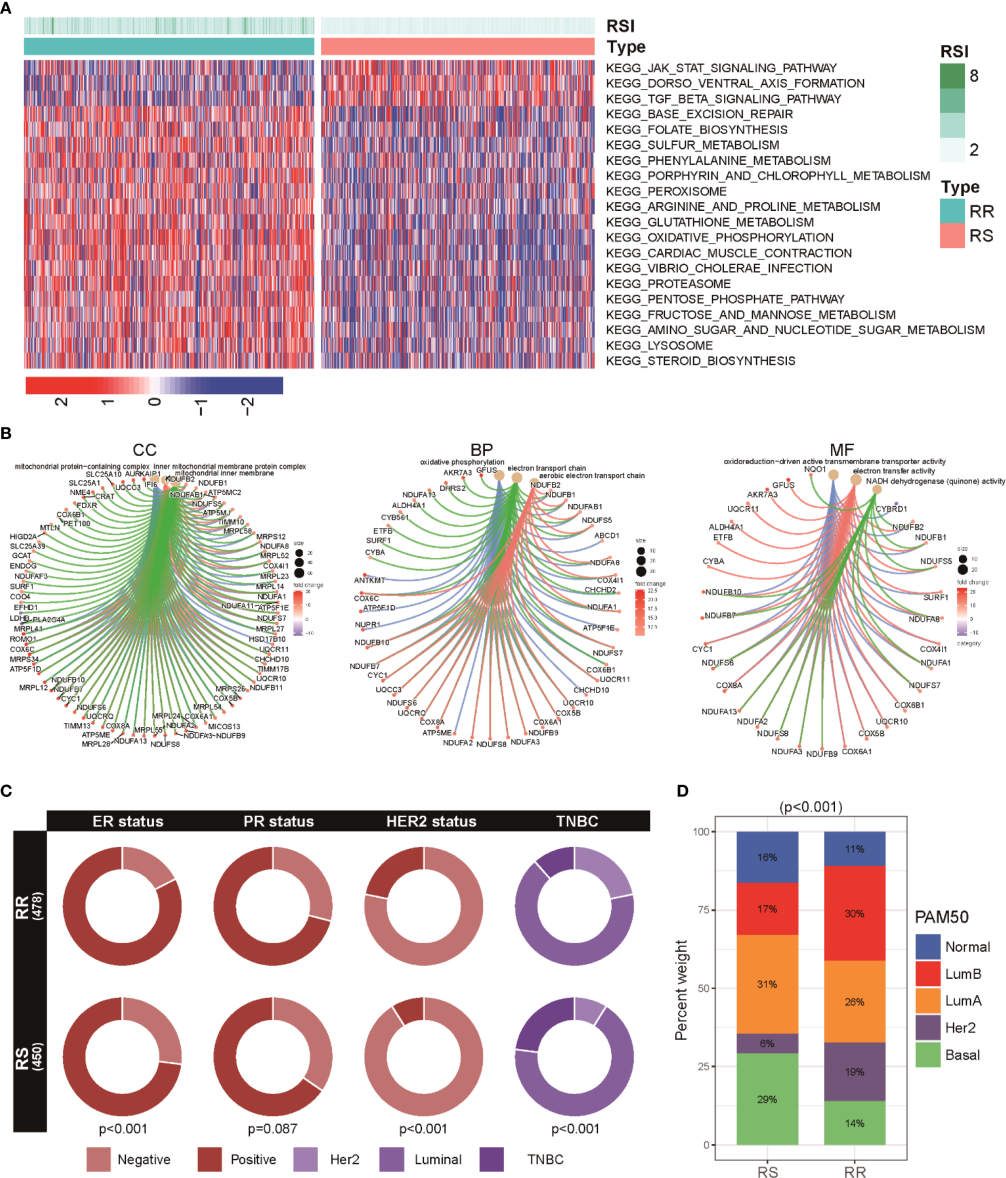
Functional enrichment and subtype distribution in radiosensitivity groups within BRCA patients. **(A)** Heatmap illustrating KEGG pathway enrichment scores for the top signaling and metabolic pathways differentially active between two groups. **(B)** GO enrichment visualization showing BP, C), and MF enriched among genes differentially expressed between RS and RR groups. **(C)** Circos plots representing the proportion of hormone receptor (ER, PR) and HER2 positive tumors across RS and RR groups. **(D)** A stacked bar chart shows the distribution of various breast cancer PAM50 subtypes (including basal, luminal A, luminal B, HER2-enriched, and normal-like) among RS and RR patients.

To understand the biological mechanisms underlying the radiosensitivity signature, we performed pathway enrichment analyses comparing RS and RR groups. KEGG pathway analysis showed that the RS subtype was predominantly enriched in the JAK/STAT signaling and TGF-β signaling cascades. In contrast, the RR group demonstrated a higher activity of metabolic pathways such as oxidative phosphorylation, phenylalanine, and arginine and proline metabolism (**Figure 3A**). Additionally, GO terms reflected distinct functional preferences: the RR group was mainly associated with processes involving positive regulation of cell activation and cell adhesion, whereas the RS group presented stronger enrichment in chromatin remodeling and DNA packaging complex-related pathways (**Figure 3B**). These results imply that the radiosensitivity signature distinguishes patients based on variations in metabolic functions, which could influence their response to radiotherapy. We also compared the distribution of hormone receptor status and HER2 expression across the two groups. Notably, RR tumors were more likely to be estrogen receptor (ER) and HER2 positive compared to RS groups (**Figure 3C**). Subtype analysis using the PAM50 classification revealed a predominance of Luminal A, Luminal B, and HER2-enriched categories in RR patients, whereas the Basal-like and Normal-like subtypes were more common among RS individuals (**Figure 3D**).

### Genomic alteration landscape in relation to radiosensitivity groups

**Figure 4:**
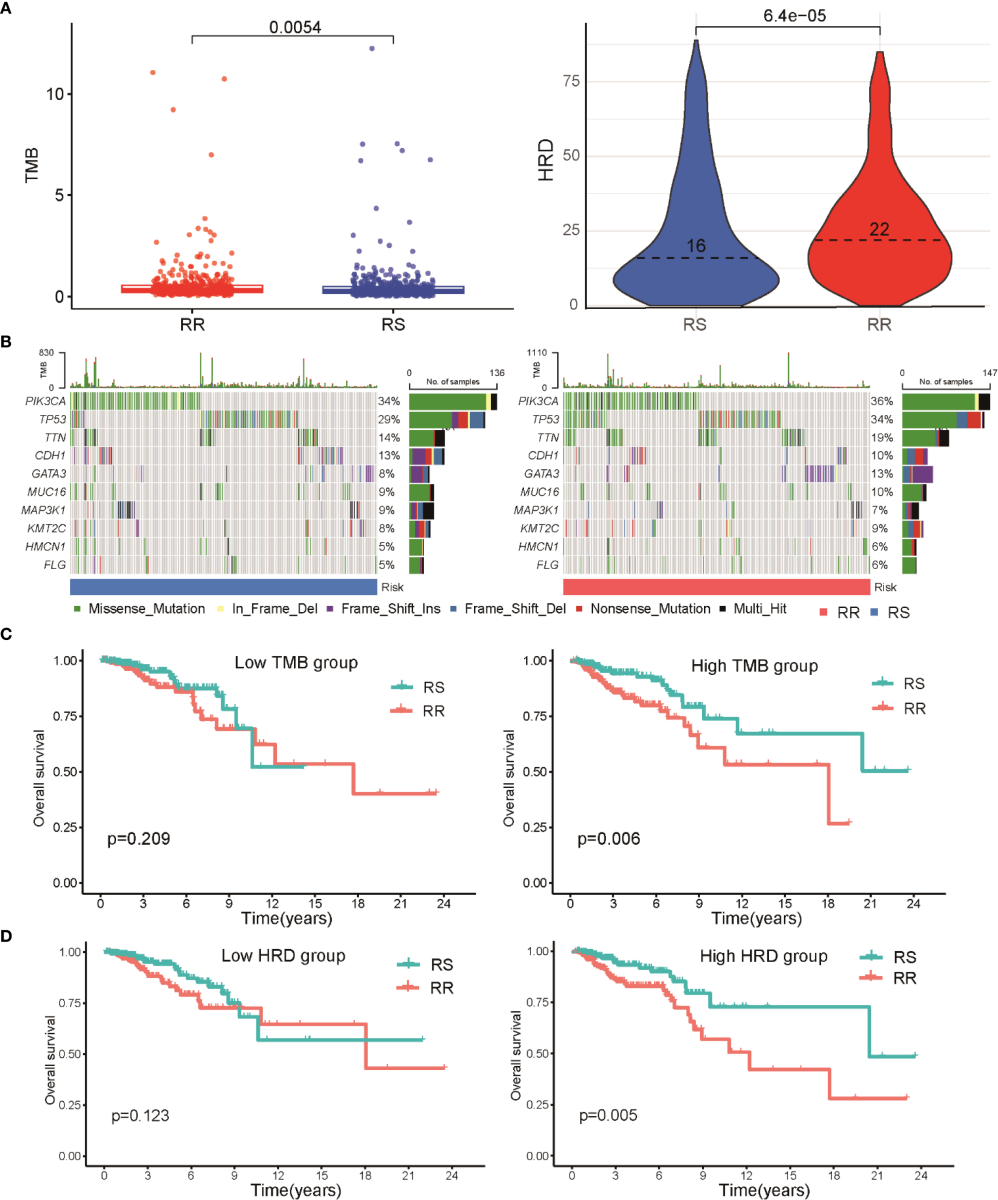
Relationship between radiosensitivity classification and genomic mutation characteristics in BRCA patients. **(A)** A scatter plot illustrates the distribution of TMB and HRD scores between RS and RR groups. **(B)** Waterfall diagrams depicting the most frequently mutated genes in RS and RR groups, with mutation types color-coded; missense mutations are predominant, and mutations in PIK3CA and TP53 are more common in the RR group. **(C)** Kaplan-Meier survival curves illustrating OS differences between RS and RR patients within high and low TMB subgroups. **(D)** Kaplan-Meier survival curves demonstrate the differences in OS between RS and RR patients based on HRD scores, indicating a poorer prognosis for RR patients with high HRD scores.

To investigate the link between radiosensitivity subtypes and genomic alterations, we analyzed tumor mutational burden (TMB) and homologous recombination deficiency (HRD) scores in both RS and RR groups. The RR subset exhibited notably elevated TMB and HRD values compared to the RS patients, indicating a correlation between higher mutation load, greater genomic instability, and radio-resistance (**Figure 4A**). A detailed mutational spectrum was constructed highlighting the top 10 genes with the highest mutation frequencies within each group. Missense mutations dominated across both cohorts, with PIK3CA and TP53 alterations occurring more frequently in RR tumors, suggesting their potential role in resistance mechanisms (**Figure 4B**). Survival analysis stratified by mutational burden showed that within the high TMB subgroup, patients classified as RS had significantly prolonged overall survival relative to their radioresistant counterparts. However, among patients with lower TMB, survival differences between RS and RR groups were not statistically significant (**Figure 4C**). A similar trend was observed in the HRD-stratified subgroup analysis. Patients in the RR group with high HRD scores exhibited a poorer prognosis when compared to those in the RS group (**Figure 4D**).

### Immune cell composition and correlations in RS and RR groups

**Figure 5:**
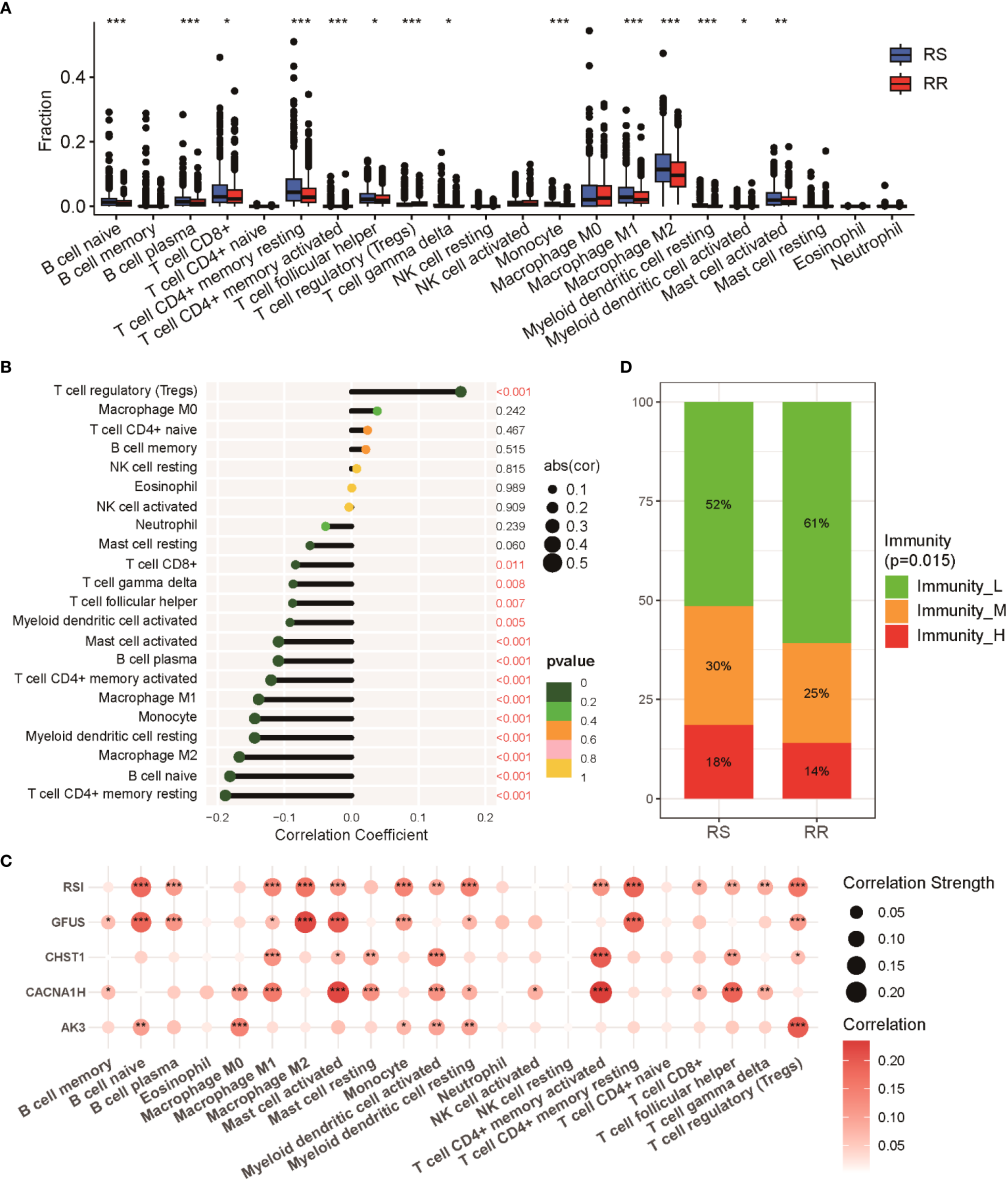
Immune cell infiltration profiles in RS and RR groups. **(A)** Relative proportions of 22 immune cell subsets within tumors of RS and RR patients as estimated by CIBERSORT. **(B)** Correlation plot depicting the relationships between various immune cell populations and the radiosensitivity index. **(C)** Examination of showing the correlations between radiosensitivity signature genes and immune cell infiltration levels. **(D)** Proportion of patients categorized into distinct immune activity clusters (high, medium, low immunity) within RS and RR groups. * p<0.05, ** p<0.01, *** p<0.001.

The tumor immune microenvironment (TIME) has been increasingly recognized as a key factor influencing response to radiotherapy (29, 30). To explore differences in immune cell composition between RS and RR groups, we applied CIBERSORT analysis to quantify tumor-infiltrating immune cells. The RS group showed elevated levels of CD8 T cells, follicular helper T cells, and both M1 and M2 macrophage populations. Conversely, the RR group was marked by a greater proportion of regulatory T cells (Tregs) (**Figure 5A**). Correlation analyses further demonstrated a notable inverse relationship between the radiosensitivity index and multiple aforementioned immune cells (**Figure 5B**). Further, relationships between the expression of individual genes in the radiosensitivity signature and immune cell abundance revealed distinct interaction patterns (**Figure 5C**). Notably, a substantial fraction of samples in the RR group were classified into the low-immunity cluster, contrasting with the RS group (**Figure 5D**). These findings provide mechanistic insights into radiation responsiveness mediated through cytotoxic immune potentiation and immunosuppression abrogation.

### Impact of the radiosensitivity signature on anti-tumor therapies

**Figure 6:**
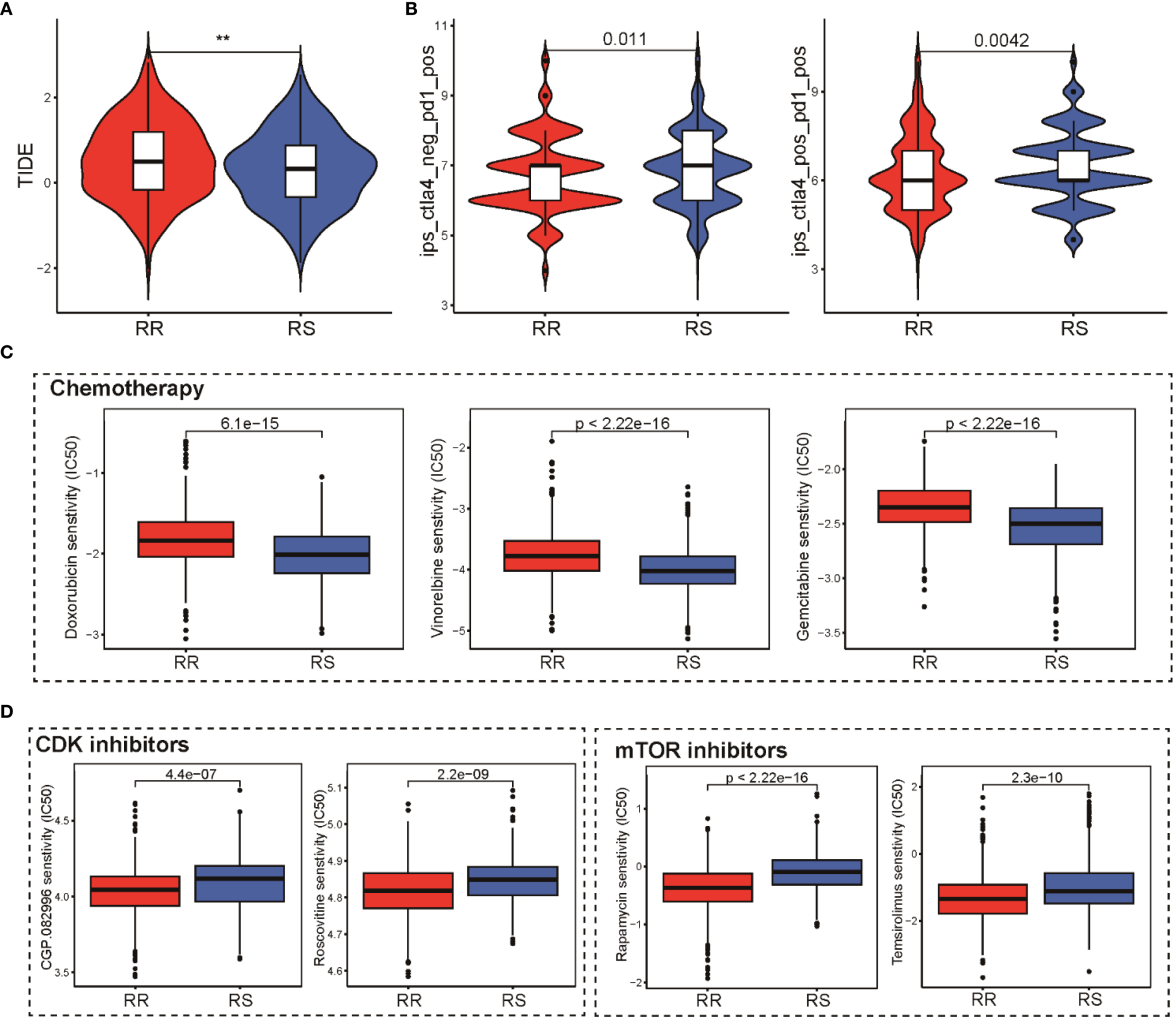
Impact of the radiosensitivity signature on various anti-tumor therapies. **(A)** Comparison of TIDE scores reflecting immune evasion probability in RS and RR groups, lower scores in RS suggest potential for superior immunotherapy outcomes. **(B)** Violin plot presenting IPS scores for PD-1 and CTLA-4 inhibitors in RS and RR groups, validating immunotherapy responsiveness. **(C)** Box plots comparing the IC50 values of doxorubicin, vinorelbine, and gemcitabine between the RR and RS groups, highlighting greater sensitivity in the RS group. **(D)** Box plots depicting the IC50 values for mTOR and CDK inhibitors in the RR and RS groups, revealing potential resistance in the RR group. ** p<0.01.

To evaluate how the radiosensitivity profile might influence therapeutic outcomes, we analyzed its association with various cancer treatment modalities, including immunotherapy, chemotherapy, and targeted agents. Using the TIDE algorithm, our analysis demonstrated that patients in the RS group exhibited significantly lower TIDE scores and exclusion scores compared to the RR group, suggesting enhanced immunotherapy response potential (**Figure 6A**). This finding was further validated using the IPS algorithm, which indicated that RS group patients showed greater responsiveness to both PD-1 and CTLA-4 inhibitors (**Figure 6B**). This suggests a higher likelihood of favorable immunotherapy outcomes in the RS group. Chemotherapy sensitivity was assessed by comparing estimated drug IC50 values between groups. Notably, the RS patients appeared more susceptible to commonly used frontline chemotherapeutic agents such as doxorubicin, vinorelbine, and gemcitabine, indicated by higher IC50 values in the RR patients (**Figure 6C**). Regarding targeted therapies, our analysis uncovered notable differences in sensitivity to mTOR and CDK inhibitors. Contrary to expectations, the RR group demonstrated lower IC50 values for these targeted agents, highlighting potential avenues to counteract radio-resistance by combining these targeted agents (**Figure 6D**). Collectively, these insights underscore the potential utility of integrating radiosensitivity signatures with therapy selection to optimize personalized treatment strategies in breast cancer.

### Prognostic and treatment value of integrating radiosensitivity with PD-L1 status

**Figure 7:**
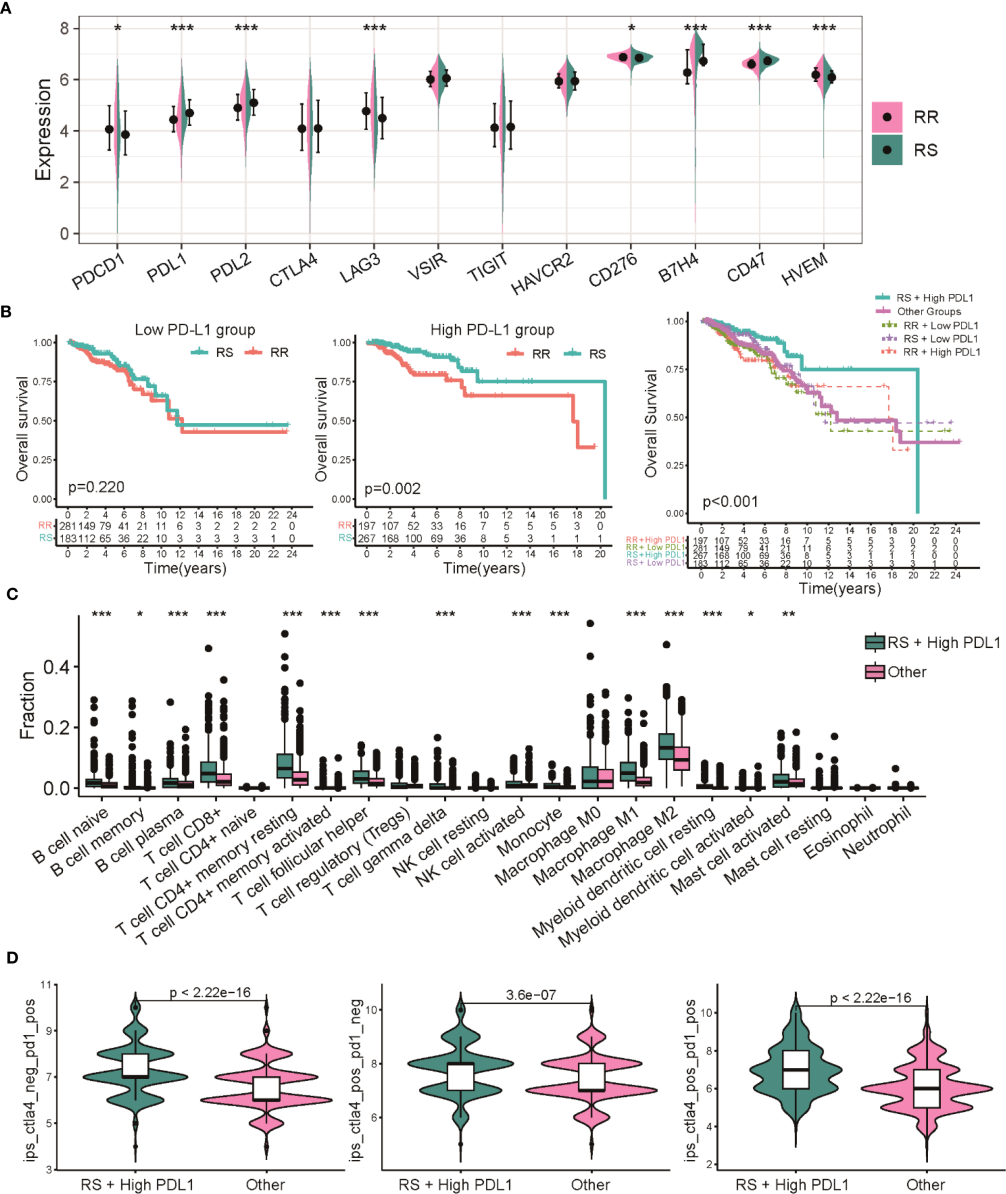
Dual biomarker stratification of therapeutic vulnerabilities in BRCA. **(A)** Comparison of expression profiles for multiple immune checkpoint genes between the RS and RR groups. **(B)** Kaplan-Meier survival analysis comparing overall survival among patients with combined radiosensitivity and PD-L1 status. **(C)** Analysis of 22 distinct immune cell infiltrate proportions between the RS High-PDL1 group and other subgroups. **(D)** Violin plots depicting the distribution of IPS scores predicting responses to CTLA-4 and PD-1 inhibitors. * p<0.05, ** p<0.01, *** p<0.001.

To further dissect how radiosensitivity intersects with immune checkpoint regulation, we assessed the prognostic significance of PD-L1 expression within the radiosensitivity-defined groups. Our findings revealed a general increase in immune checkpoint genes, notably PD-L1, in the RS group relative to the RR group (**Figure 7A**). However, when considering PD-L1 expression alone or its interaction with radiotherapy, no significant differences in OS were observed (**Supplementary Figure S5A**). However, stratification combining radiosensitivity and PD-L1 status revealed that patients in the RS group with high PD-L1 expression exhibited significantly better overall survival compared to the RR group. Conversely, no significant survival difference was found between RS and RR patients with low PD-L1 levels. This suggests that patients classified as RS with elevated PD-L1 expression (RS High-PD-L1) experienced a markedly improved OS compared to other subgroups (**Figure 7B**). ESTIMATE analysis showed significant stromal, immune scores and estimate score in the RS-PD-L1-high subgroup compared to other groups (**Supplementary Figure S5B**). Further analysis using CIBERSORT revealed an elevated infiltration of CD8 T cells, CD4 T cells, and other immune cell types within the tumor immune microenvironment of the RS High-PDL1 group, indicating a more immune-responsive tumor microenvironment (**Figure 7C**). To predict immunotherapy efficacy, IPS scoring was applied, indicating enhanced potential response to PD-1 and CTLA-4 inhibitors within this RS High-PD-L1 group (**Figure 7D**). These observations highlight that integrating radiosensitivity signatures with PD-L1 expression may refine patient stratification and better predict immunotherapy outcomes in breast cancer.

### *In vitro* validation of radiosensitivity signature and glycolysis in breast cancer cells

**Figure 8:**
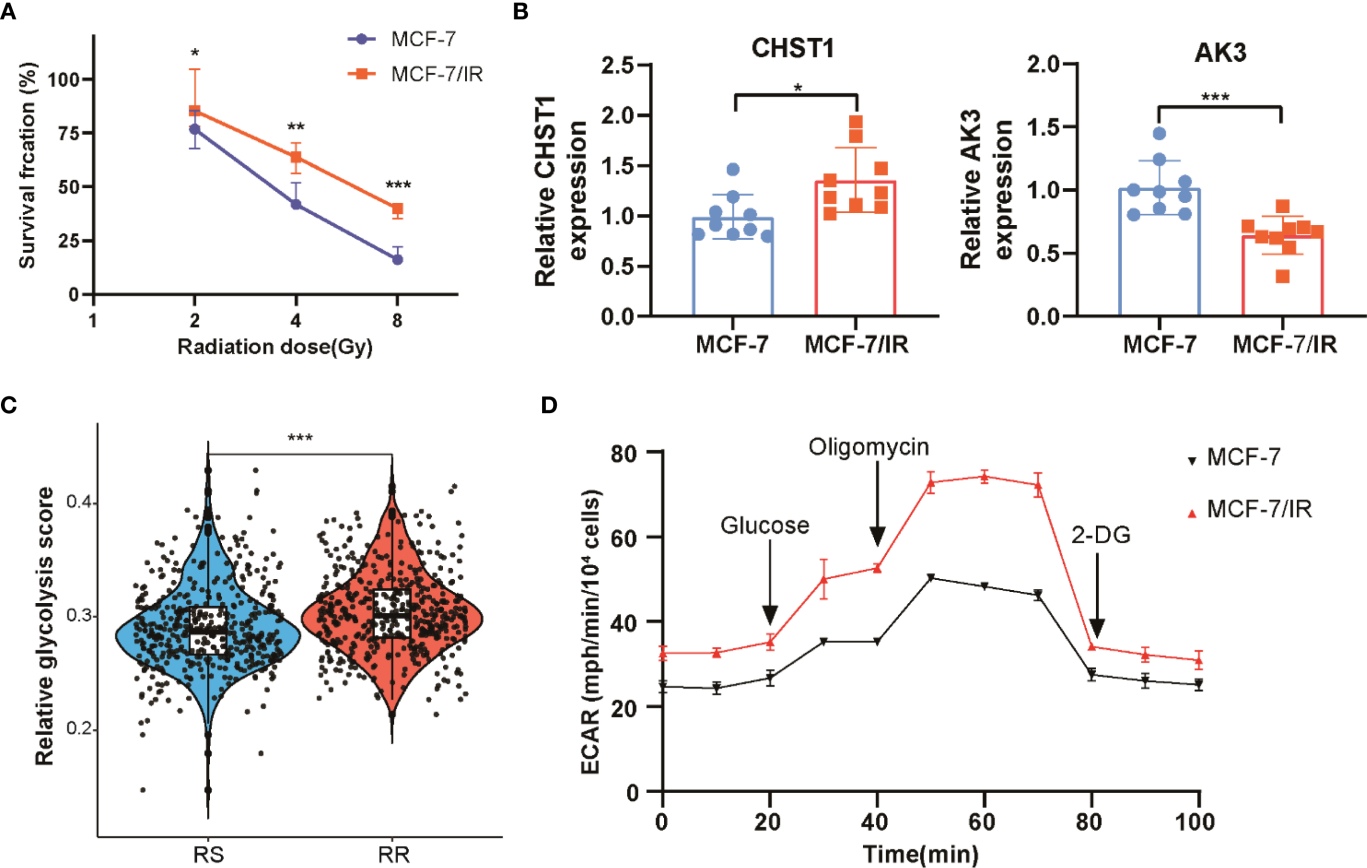
*In Vitro* assessment of radiosensitivity and glycolytic activity in breast cancer cell. **(A)** Cell viability curves comparing proliferation of MCF-7 and MCF-7/IR cells after various doses of ionizing radiation. **(B)** Quantitative RT-PCR analysis of CHST1 and AK3 mRNA levels in MCF-7 versus MCF-7/IR cells. **(C)** Scatter plot illustrating the distribution of glycolysis scores in RS and RR groups, derived from ssGSEA analysis. **(D)** Seahorse extracellular acidification rate (ECAR) profiles monitoring glycolytic activity over time in MCF-7 and MCF-7/IR cells. ** p<0.01. *** p<0.001.

To validate the radiosensitivity signature derived from computational analysis, we conducted *in vitro* experiments using MCF-7 and its radioresistant derivative MCF-7/IR cell lines. Cell viability assays following exposure to increasing doses of ionizing radiation demonstrated that MCF-7/IR cells exhibited greater viability compared to MCF-7 cells, consistent with their radio-resistant phenotype (**Figure 8A**). Gene expression analysis by qRT-PCR confirmed upregulation of CHST1 and downregulation of AK3 in the radioresistant MCF-7/IR cells, consistent with bioinformatics findings (**Figure 8B**). Glycolysis plays a crucial role in tumor energy metabolism and radio-resistance. We observed elevated glycolysis scores in the RR group compared to the RS group via ssGSEA analysis in TCGA-BRCA dataset (**Figure 8C**). Extending this observation to cellular metabolism, ECAR measurements via Seahorse assay revealed heightened glycolytic flux in MCF-7/IR cells compared to MCF-7, indicating increased glycolytic activity in MCF-7/IR cells (**Figure 8D**). Together, these data provide experimental support linking altered expression of radiosensitivity genes and enhanced glycolysis to radiation resistance in breast cancer.

## Discussion

The present study aimed to explore the predictive value of glycolytic activity in determining radiotherapy efficacy for BRCA patients and to develop a radiosensitivity signature associated with glycolysis. By categorizing BRCA patients into low-glycolysis and high-glycolysis groups, we found that glycolytic activity correlated with reduced radiotherapy efficacy and poor survival outcomes. We then constructed a radiosensitivity signature incorporating four glycolysis-related genes, which successfully stratified patients into RS and RR groups, highlighting the potential role of glycolytic activity as a biomarker for treatment efficacy in breast cancer. Further analyses revealed distinct differences in clinicopathological features, functional pathways, genomic alterations, immune cell composition, and therapeutic responses between the RS and RR groups. By establishing the relationship between glycolytic activity and radiotherapy efficacy, we provide a compelling argument for the incorporation of metabolic profiling into clinical practice.

The observed relationship between glycolytic activity and radiotherapy outcomes underscores the significance of metabolic reprogramming in cancer cell behavior and treatment responses (31). Our study supports existing literature that suggests patients in the low-glycolysis group exhibit enhanced sensitivity to radiotherapy (32). Recent pan-cancer analyses have highlighted the critical role that pathways governing cell cycle progression, such as those involving mitotic DNA integrity checkpoint kinases, play in determining cancer cell fate (33). This increased sensitivity may be attributed to several mechanisms, including enhanced apoptosis pathways and a less immunosuppressive tumor microenvironment found in low-glycolysis tumors (34). Conversely, tumors characterized by high glycolytic activity often present an aggressive phenotype, frequently associated with radio-resistance due to mechanisms such as increased DNA repair capacity, enhanced cellular proliferation, and evasion of apoptosis influenced by metabolic byproducts (35). Additionally, our RSI, which includes key genes like AK3, CACNA1H, CHST1, and GFUS, offers a novel framework for predicting treatment outcomes. Briefly, the four genes comprising the signature have established biological functions that may influence radiotherapy response. AK3 is a mitochondrial enzyme crucial for energy homeostasis and nucleotide metabolism; its downregulation in radioresistant tumors, as suggested by our model, may disrupt cellular energy balance and impair DNA damage repair pathways (36). CACNA1H encodes a T-type calcium channel. Aberrant calcium signaling is known to be involved in cancer progression and treatment resistance, potentially by modulating cell proliferation and apoptosis pathways following DNA damage (37). CHST1 is involved in modifying glycoproteins and glycolipids. Its upregulation could alter cell adhesion and signaling within the tumor microenvironment, promoting a more aggressive and treatment-refractory phenotype (38). GFUS is a key enzyme in fucose metabolism and protein fucosylation. Altered fucosylation is increasingly recognized as a modulator of tumor growth, immune evasion, and resistance to therapy, suggesting its role in modulating the cellular response to radiation (39). Collectively, the RSI serves as more than a predictive algorithm; it represents a convergence of key biological pathways that mechanistically drive radiation sensitivity in breast cancer.

The interplay between the tumor immune microenvironment and radiotherapy response has garnered significant attention in recent oncology research (8, 40). Our results demonstrated distinct differences in immune cell composition between the RS and RR groups. These observations align with prior research emphasizing the association between “hot” tumor phenotypes and favorable responses to immunotherapy and radiotherapy (41). The elevated levels of CD8 T cells and follicular helper T cells in the RS group suggest a more active anti-tumor immune response. Interestingly, we observed significantly higher expression of the macrophage checkpoint CD47 in RS tumors. While this seems paradoxical for a “don’t eat me” signal, we hypothesize that this high dependency on CD47 for immune evasion represents a key vulnerability (42). Radiotherapy-induced “eat me” signals could overwhelm this protection, leading to enhanced macrophage-mediated clearance in tumors reliant on the CD47 axis. This model suggests that high baseline CD47 may, in fact, predict synergistic responses to combined radiotherapy and CD47 blockade. Conversely, the RR group enrichment in Tregs suggests an immunosuppressive milieu that could mitigate the effects of radiotherapy, as Tregs are known to inhibit effector T cell function and promote tumor progression (43). This is consistent with studies showing that Treg expansion correlates with poor prognosis in breast cancer and reduced efficacy of immune checkpoint inhibitors (44). Moreover, our correlation analyses reveal an inverse relationship between the radiosensitivity index and immune cell abundance, emphasizing that higher glycolytic activity may correlate with a less favorable immune landscape. This suggests that integrating immune profiling with metabolic status could enhance our understanding of tumor biology and therapeutic efficacy.

In terms of therapeutic strategies, the radiosensitivity profile notably impacts responses to various treatment modalities, including immunotherapy and chemotherapy (45). The enhanced immunotherapy responsiveness in the RS group, as indicated by lower TIDE and IPS scores, aligns with emerging clinical data showing that tumors with pre-existing T cell inflammation exhibit superior anti-PD-1 responses (46, 47). Interestingly, the RR group paradoxical sensitivity to mTOR and CDK inhibitors highlights a potential metabolic dependency in radioresistant tumors. This is consistent with emerging evidence linking metabolic reprogramming, such as upregulated glycolysis, to radiation resistance, where targeting metabolic pathways may synergize with conventional therapies (48). Notably, the lower IC50 values for mTOR/CDK inhibitors in the RR group suggest that these agents could be leveraged to counteract radio-resistance, a strategy warranting further exploration in preclinical and clinical settings. Furthermore, the enhanced OS seen in the RS High-PD-L1 group suggests that metabolic profiles and immune checkpoint expression interact synergistically to influence tumor responsiveness to therapy. These findings align with the notion that patients with favorable metabolic profiles may also benefit from immunotherapeutic strategies (49), emphasizing the integration of radiosensitivity with PD-L1 status represents a promising step toward precision medicine in breast cancer.

Looking forward, our findings could pave the way for clinical translation. The glycolysis-related radiosensitivity signature we have developed could be engineered into a practical molecular test to stratify breast cancer patients. One could imagine a clinical assay, based on tumor biopsies or even liquid biopsies, that measures the expression of AK3, CACNA1H, CHST1, and GFUS. This would identify patients with high predicted glycolytic activity who are less likely to respond to standard radiotherapy. Such patients might then be candidates for intensified radiation regimens or, perhaps more promisingly, the co-administration of radio-sensitizing agents. Several metabolic inhibitors targeting glycolytic pathways are already under investigation and could be rationally combined with radiotherapy to overcome resistance in the RR group. Furthermore, advances in computational biology offer powerful tools to refine such predictive models (50, 51). This technique could augment our training dataset, helping to improve the signature’s robustness and predictive accuracy across diverse and underrepresented patient populations, thereby facilitating the development of a more reliable clinical test.

Despite the promising results, this study has several limitations. First, the reliance on retrospective analyses from public databases like TCGA and METABRIC may introduce biases related to treatment variations and patient heterogeneity. Second, the methodology employed for categorizing patients based on glycolytic activity requires further validation in prospective studies. Additionally, while the sample size is relatively large, variations in clinical parameters, it may not capture the full spectrum of breast cancer heterogeneity. Moreover, our *in vitro* validation was limited to a single isogenic cell line pair, and while this provided a controlled proof-of-concept, broader experimental validation across a diverse panel of preclinical models is essential to confirm the generalizability of our findings. Lastly, the functional mechanisms underlying the radiosensitivity signature and its interaction with the tumor immune microenvironment warrant further investigation. Experimental validation of the radiosensitivity signature in various preclinical models would strengthen the applicability of these findings.

## Conclusion

In conclusion, our study establishes glycolytic activity as a promising predictor of radiotherapy efficacy in BRCA patients. We developed a glycolysis-related radiosensitivity signature that enhances patient stratification and improves clinical management by linking glycolytic activity to treatment outcomes. These findings underscore the importance of considering tumor glycolysis and associated biological processes when designing personalized radiotherapy strategies. Future research should focus on validating these results in prospective cohorts and exploring the therapeutic implications of targeting glycolysis. This approach may ultimately lead to more effective treatments and improved outcomes for BRCA patients undergoing radiotherapy, highlighting the need for further exploration of the tumor microenvironment and metabolic factors in cancer therapeutics.

## Data Availability

The data used in this study are derived from the Molecular Taxonomy of Breast Cancer International Consortium (METABRIC) via the cBioPortal (https://www.cbioportal.org).
