# Supplementary material for: Glycolysis-related radiosensitivity signature for predicting radiotherapy response in breast cancer
Source: Front Immunol. 2025 Oct 2;16:1638897. doi: 10.3389/fimmu.2025.1638897 (PMC12529102; doi:10.3389/fimmu.2025.1638897)
Supplement: Supplementary file 1 [file DataSheet1.docx]

**Supplement Figures**

**
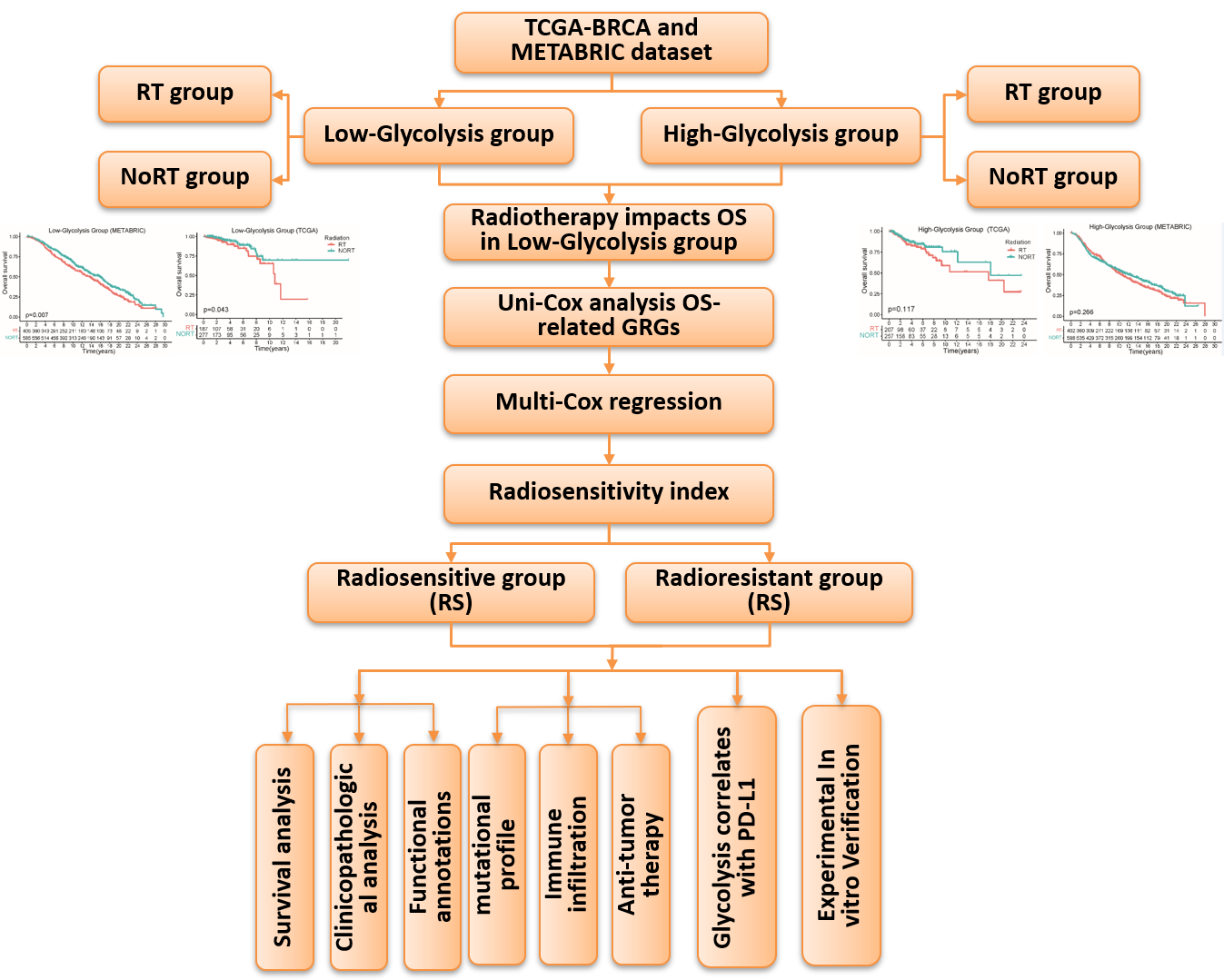
**

**Figure S1** The methodological pipeline of our study.


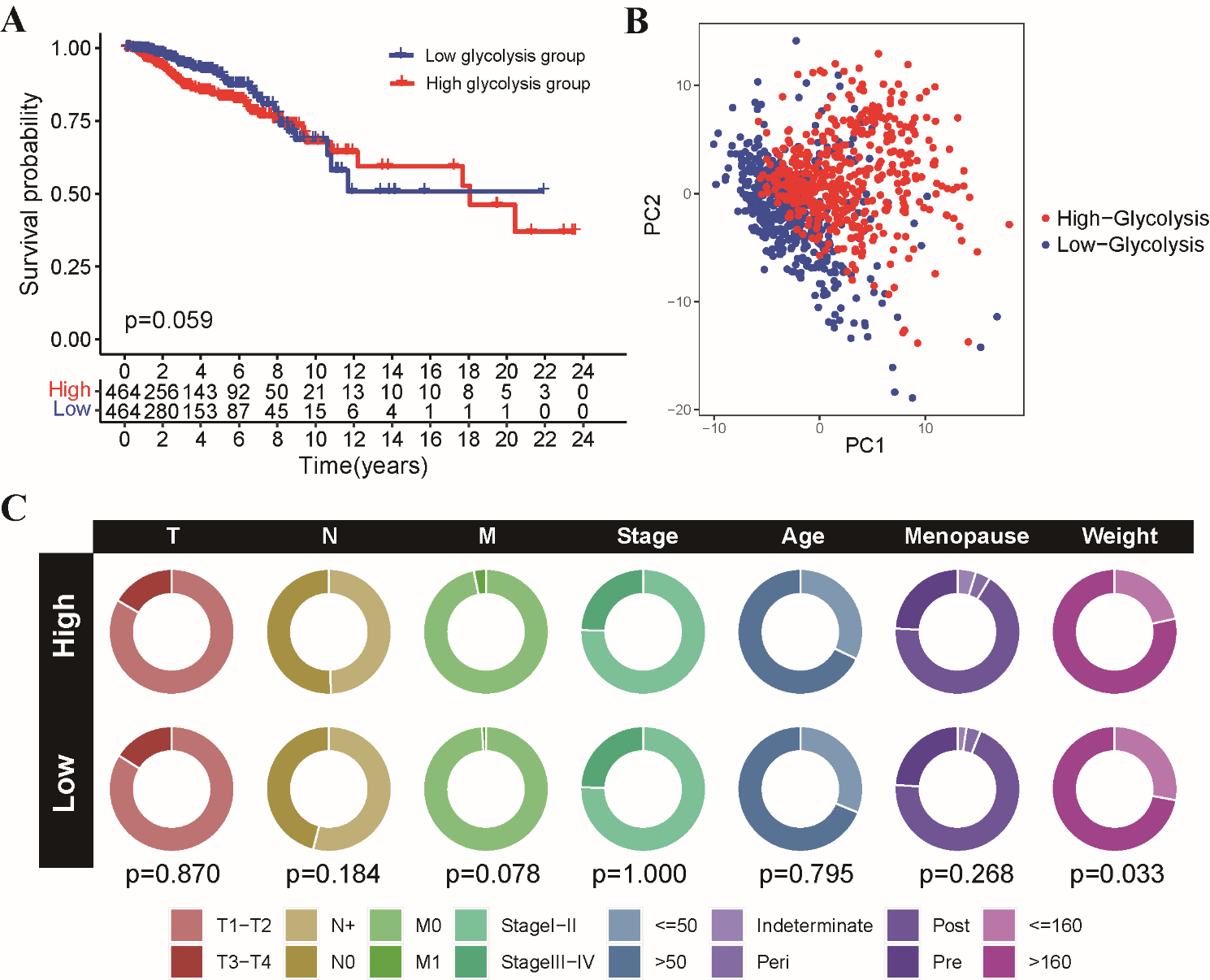


**Figure S2 (A)** Kaplan-Meier survival analysis depicting overall survival (OS) differences between patients with high and low glycolytic activity. **(B)** Principal component analysis performed on the expression profiles of 200 glycolysis-associated genes from the TCGA dataset. **(C)** Summary of the association between glycolytic activity and clinicopathological characteristics in individuals diagnosed with BRCA.

**
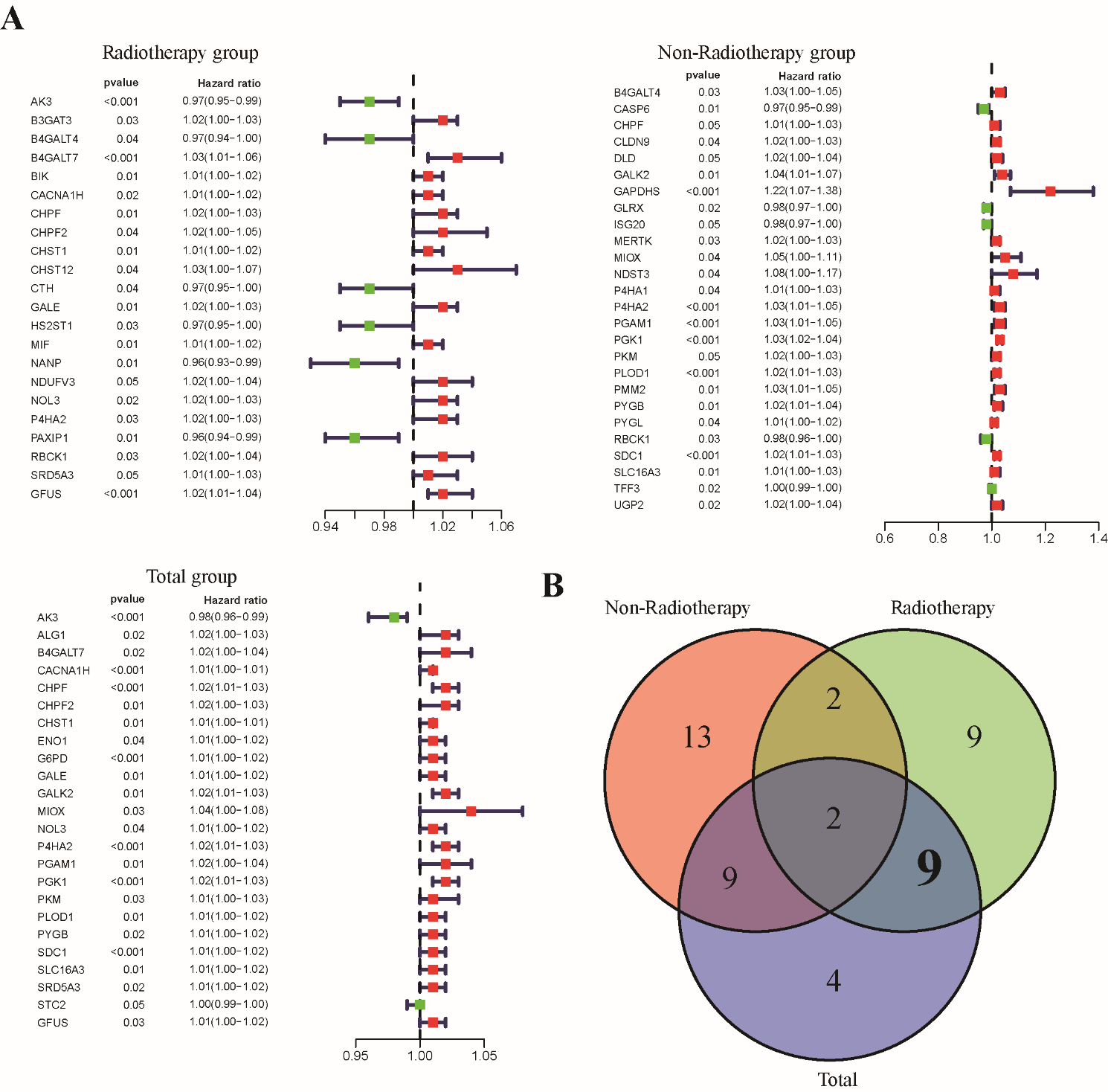
**

**Figure S3** **(A)** Forest plot illustrating the outcomes of univariate Cox proportional hazards analysis in patients receiving radiotherapy, those not receiving radiotherapy, and the entire patient cohort. **(B)** A Venn diagram highlighting the overlap of glycolysis-related genes significantly linked to OS, where 9 genes were found to be significant in both the radiotherapy and total patient groups, but not in the non-radiotherapy group.


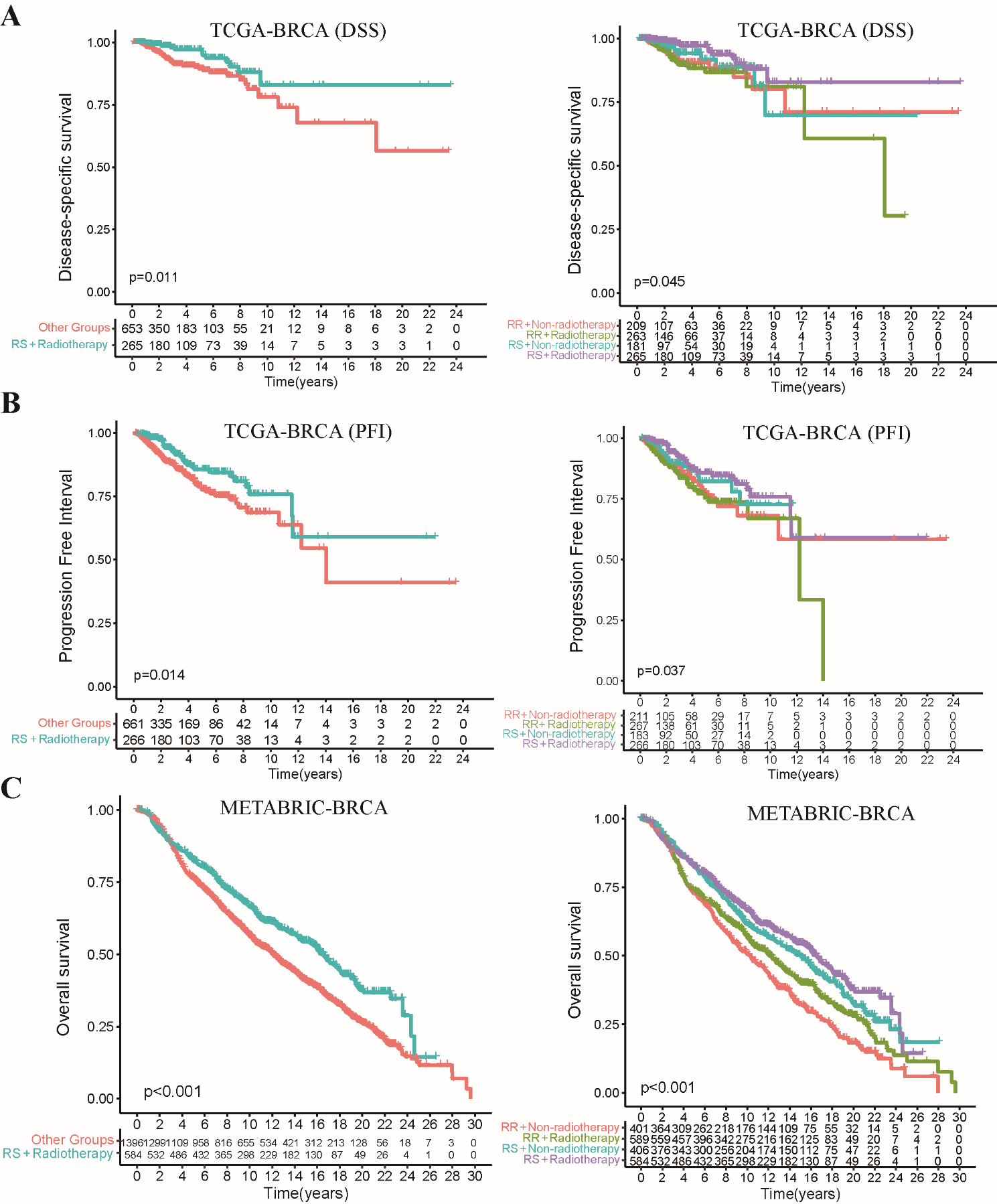


**Figure S4** **(A)** Kaplan-Meier survival analysis illustrating the difference in DSS among radiotherapy patients classified in the RS group versus those in all other categories. **(B)** Kaplan-Meier estimates of PFI for radiotherapy patients according to their classification in the RS group compared with non-RS patients. **(C)** Kaplan-Meier survival curves displaying OS in the METABRIC cohort stratified by both radiosensitivity status (RS vs. RR) and whether patients received radiotherapy.

**
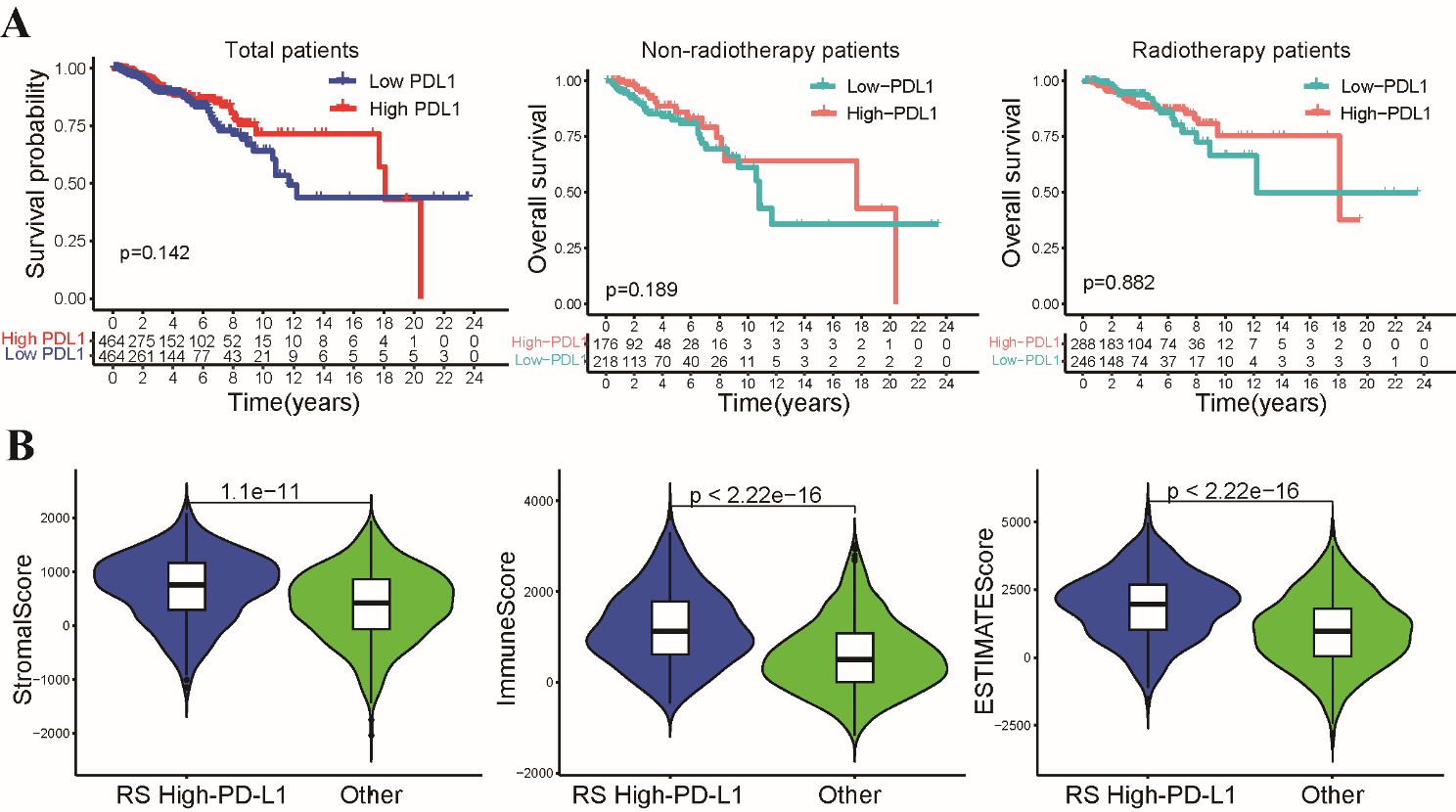
**

**Figure S5** **(A)** Kaplan-Meier curves showing overall survival stratified by radiotherapy receipt across PD-L1 expression groups. **(B)** Violin plots comparing estimated, stromal, and immune scores between the PD-L1-high-RR group and other subgroups.

**Table S1 |** Sequences of the primer used for qRT-PCR

| mRNA |  | Forward primer | Reverse primer |
| --- | --- | --- | --- |
| AK3 |  | ACGCCTTACTGCTCGCTGGATT | CTCACGCTGAATGAGAGGCTCC |
| CHST1 |  | CGTCTTCTACCTGTTTGAGCCC | GGAAGTAGAGGTCGCAGTCGTA |
| GFUS |  | AAGCCATCCAGAAGGTGGTAGC | GGTTGGACCTTCTCAAACAGGG |
| CACNA1H |  | GGAACATCTCCACCAAGGCACA | TCCATCCTTGGATGACAGCACG |
| GAPDH |  | GTCTCCTCTGACTTCAACAGCG | ACCACCCTGTTGCTGTAGCCAA |

The primer sequences used in this study were obtained from OriGene Technologies, Inc.

AK3 Human qPCR Primer Pair (NM_005201)，CAT#: HP208379

CHST1 Human qPCR Primer Pair (NM_019846)，CAT#: HP213463

GFUS Human qPCR Primer Pair (NM_002089)，CAT#: HK202326

CACNA1H Human qPCR Primer Pair (NM_002089)，CAT#: HK202326

GAPDH Human qPCR Primer Pair (NM_002046)，CAT#: HP205798
